# Vitamin A in children’s pneumonia for a COVID-19 perspective: A systematic review and meta-analysis of 15 trials

**DOI:** 10.1097/MD.0000000000031289

**Published:** 2022-10-21

**Authors:** Ruoxi Li, Wenli Zhao, Hongwu Wang, Maeda Toshiyoshi, Ye Zhao, Huaien Bu

**Affiliations:** a Graduate School, Shandong University of Traditional Chinese Medicine, Jinan, China; b Liver Center, Saga University Hospital, Saga University, Saga, Japan; c School of Health Science and Engineering, Tianjin University of Traditional Chinese Medicine, Tianjin, China; d International Education College, Shandong University of Traditional Chinese Medicine, Jinan, China; e Department of Public Health, International College, Krirk University, Bangkok, Thailand.

**Keywords:** children, meta-analysis, pneumonia, randomized controlled trials, systematic review, vitamin A

## Abstract

To systematically review and meta-analyze the efficacy of vitamin A as an adjuvant therapy for pneumonia in children.

**Methods:**

We searched in PubMed, the Cochrane Library, Chinese National Knowledge Infrastructure, WanFang Database and Chongqing VIP information network from libraries building to March 2022, screening randomized controlled trials (RCT) about vitamin A combined with conventional therapy for pneumonia in children. Two researchers used the Cochrane risk of bias tool to assess the quality of included studies dependently. Data analysis was conducted in the RevMan 5.3.

**Results:**

15 trials involving 3496 patients (treated group: 1898; control group: 1598) were analyzed in this study. The Meta-analysis showed that vitamin A combined with conventional therapy improved clinical efficacy (*P* < .05), shortened the duration of fever and cough, negative time of chest X-ray, and the hospitalization, lung rale disappearance, choking milk disappearance, shortness of breath disappearance and perilabial cyanosis disappearance (*P* < .05). However, vitamin A combined with conventional therapy did not reduce the mortality of pneumonia in children (*P* > .05).

**Conclusion:**

Vitamin A contributes to relieve the clinical symptoms and signs, and also shorten the hospitalization.

## 1. Introduction

Pneumonia is a common disease among children.^[1]^ Children are vulnerable to various syndromes and even death due to their young age and poor immunity.^[2]^ According to the World Health Organization, 20% of deaths among children aged <5 years were caused by pneumonia.^[3]^ In 2015, an estimated 921,000 children younger than 5 years died of pneumonia.^[4]^ About 1.6 million newborns die from pneumonia worldwide each year.^[5]^ It shows that 81% of children with pneumonia had pathogens. Viral pathogens are detected in 73% of the children and bacterial pathogens in 15%. Among the viral pathogens, respiratory syncytial virus is the most common pathogen detected.^[6]^ *Streptococcus pneumoniae* is the most common cause of bacterial pneumonia in children. There are also a small number of fungal pneumonias. The clinical symptoms for pneumonia in children are mainly fever, refusal of food, fidgety, asthmatic and suffocating. In addition to respiratory symptoms, children are accompanied by mental depression, restlessness, loss of appetite, diarrhea and other systemic symptoms. Infants are mainly refusal of food, milk choking, vomiting and dyspnea.

The treatment principle of pneumonia in children mainly includes supportive care (viral pneumonia), oral antibiotics, early treatment, adequate course of treatment, and intravenous administration of severe illness.

Recent studies have found that vitamin A can participate in many aspects of immune function. It has a positive effect on body immunity by maintaining the integrity of epithelial cells, enhancing the number and activity of innate immune cells, and promoting the synthesis of immunoglobulin.^[7]^ Vitamin A deficiency (VAD) not only affects children’s visual function and growth, but also impairs immune function.^[8]^ Children are vulnerable to various diseases because of VAD.^[9,10]^ Vitamin A as an adjuvant therapy has a good effect for pneumonia in children.^[11]^ VAD is common in children with pneumonia.^[12]^ The incidence of pneumonia in children is closely related to the level of vitamin A in the serum.^[13]^ Also, vitamin A can effectively improve the treatment effect of pneumonia in children.^[14]^ However, the effect of vitamin A for pneumonia in children remains controversial. Therefore, we conducted a systematic review to investigate the efficacy of vitamin A for pneumonia in children.

## 2. Methods

### 2.1. Search strategy

We performed a comprehensive literature search in PubMed, the Cochrane Library, Chinese National Knowledge Infrastructure, WanFang Database and Chongqing VIP information network from libraries building to March 2022, using the combination of subject word and free word. The following search words were used: pneumonia, pneumon, pulmonary inflammat, retinoid, vitamin A, pulmonary Infect, lung Infect, immunity, child, children, and kid.

### 2.2. Criteria for inclusion and exclusion

#### 2.2.1 Study design

Only published randomized controlled trial (RCT) is included, no language restriction is used.

#### 2.2.2 Object of study

The object of study meets the diagnostic criteria for pediatric pneumonia, and there are no gender, race, or geographical restrictions.

#### 2.2.3 Interventions

Trials comparing vitamin A and conventional therapy with conventional therapy are included. The experimental group receives conventional treatment combined with vitamin A, while the control group only receives conventional treatment.

#### 2.2.4 Outcome indicators

Clinical efficacy, negative time of chest X-ray, the duration of fever and cough, the hospitalization, lung rale disappearance, choking milk disappearance, perilabial cyanosis disappearance or mortality rate.

### 2.3. Exclusion criteria were as follows

 Duplicated studies, animal studies, review and observational studies, unqualified studies, subjects did not meet the diagnostic criteria for childhood pneumonia, non-clinical randomized trials.

### 2.4. Data extraction and assessment of quality

We state that an ethics committee or institutional review board is not applicable in the study. Two researchers used the Cochrane risk of bias tool to assess the quality of included studies dependently. Any agreements were solved by consensus. It consists of random sequence generation, allocation concealment, binding of participants and personnel, binding of outcome assessment, incomplete outcome, selective reporting, and other bias. “+” indicates low risk of bias, “−” indicates high risk of bias, “?” indicates unclear risk of bias.

### 2.5. Statistical analysis

Statistical analysis was performed in RevMan 5.3. Continuous data was expressed as mean difference (MD) with 95% confidence interval (CI), and the dichotomous data was expressed as risk ratio (RR) with 95% CI. Heterogeneity across trials was assessed via the Chi-square test with significance being set at *P* < .10 and also assessed by means of *I*^2^. The random-effect model was used when high heterogeneity was assessed (*I*^2^ > 50% or *P* < .10); Otherwise, the fixed-effect model was used.

## 3. Results

### 3.1. Study selection

**Figure 1.**
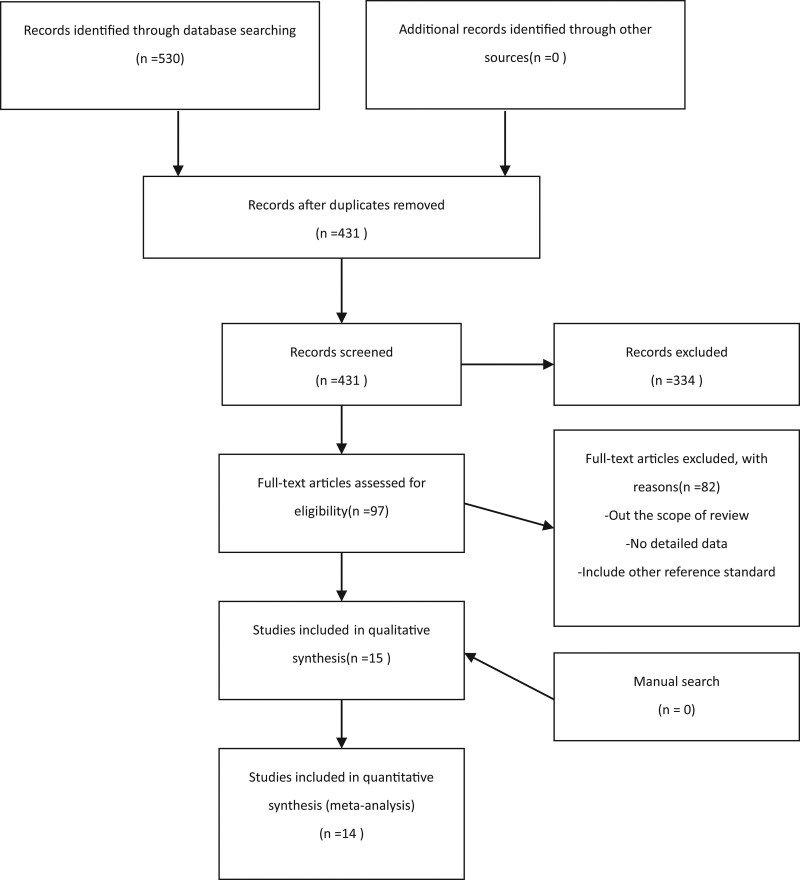
The flow diagram of study selection process.

A total of 530 studies were identified through electronic searches, and 99 studies were excluded because of duplicates removed. Then 334 studies were also excluded after reading the title and abstract. Eighty two studies were excluded by reading the full texts. And there are 15 studies included in qualitative synthesis and meta-analysis finally. The flow diagram of study selection process is shown in Figure 1.

### 3.2. Baseline characteristics and risk of bias of included studies

The baseline characteristics of included studies are summarized in Table 1. The risk of bias of included studies is graphically summarized in Table 2.

**Table 1:** Baseline characteristics of included studies.

Study	Location	Age	Study design	Sample size (treated group/control group)	The dose of vitamin A	Outcomes
Zhang 1999^[15]^	China	/	Randomized controlled	40/40	20,000 IU × 6 d to 1500 IU × 20 d	②
Rodriguez 2005^[14]^	Ecuador	2–59 mo	Double-blind, randomized control	145/142	<1 yr: 50,000 IU	①③⑤
					>1 yr: 100,000 IU	
Stephensen 1998^[16]^	Peru	3 mo–10 yr	Double-blind, randomized control	48/47	<1 yr: 50,000 IU after 100,000 IU on the first d	⑦
					>1 yr: 100,000 IU after 200,000 IU on the first d	
Si 1997^[17]^	Vietnam	1–59 mo	Double-blind, randomized control	280/312	<1 yr: 200,000 IU	①③⑦
					>1 yr: 400,000 IU	
Nacul 1997^[18]^	Brazil	6–59 mo	Double-blind, randomized control	239/233	<1 yr: 200,000 IU	①③
					>1 yr: 400,000 IU	
Fawzi 1998^[19]^	Tanzania	6–60 mo	Double-blind, randomized control	346/341	<1 yr:200,000 IU	①③⑤⑦
					>1 yr:400,000 IU	
Ma 2000^[20]^	China	/	Randomized controlled	42/36	1500 IU	③④⑤
Gu 2001^[21]^	China	/	Randomized controlled	60/60	Infants:1500 IU;	③④⑤
					Children: 2000 IU	
Wang 2003^[22]^	China	4 mo–8 yr	Double-blind, randomized control	68/40	5000 IU/(kg·d), obviously VAD after intramuscular injection 0.5–1 mL × 3 d reduced to 5000 IU/(kg·d)	③④⑤⑥⑦
Yang 2015^[23]^	China	/	Randomized controlled	41/39	5000 U × 7 d	④⑤⑧⑨
Guo 2016^[24]^	China	/	Randomized controlled	43/43	4500 IU × 7 d	③④⑤⑥
Liu 2015^[25]^	China	7–9 mo	Randomized controlled	60/60	(20,00,000 U/6.15 kg) × 7 d	④⑤⑧⑨
Yin 2017^[26]^	China	5 mo–9 yr	Randomized controlled	50/50	5000–15,000 U × 7 d	⑥
Dong 2017^[27]^	China	8–9 mo	Randomized controlled	128/128	4500 IU × 7 d	③④⑤⑦⑧
Liang 2018^[28]^	China	6–7 yr	Randomized controlled	30/30	3000–5000 U × 7 d	④⑤⑥

Notes: / = not mentioned, ① = mortality rate, ② = the clinical efficacy, ③ = the duration of fever, ④ = the duration of cough, ⑤ = the time of lung rale disappearance, ⑥ = negative time of chest X-ray, ⑦ = the time of hospitalization, ⑧ = the time of perilabial cyanosis disappearance, ⑨ = the time of choking milk disappearance.

**Table 2:** The assessment of quality of included studies.

	Random sequence generation	Allocation concealment	Binding of participants and personnel	Binding of outcome assessment	Incomplete outcome	Selective reporting	Other bias
Nacul 1997	+	+	+	+	+	+	?
Fawzi 1998	+	?	+	+	+	+	?
Stephensen 1998	+	?	+	+	+	+	?
Rodríguez 2005	+	+	+	+	+	+	?
Si 1997	+	?	+	+	?	+	?
Zhang 1999	-	-	?	?	+	?	?
Ma 2000	?	-	?	?	+	?	?
Gu 2001	?	?	?	?	?	?	?
Wang 2003	?	-	+	?	?	?	?
Yang 2015	+	-	-	-	+	+	?
Liu 2015	+	-	-	-	+	+	?
Guo 2016	+	-	-	-	+	+	?
Yin 2017	+	-	-	-	+	+	?
Dong 2017	+	-	-	_	+	+	?
Liang 2018	+	-	-	-	-	+	?

“+” = low risk, “?” = unclear, “-” = high risk.

### 3.3. Overall outcomes

#### 3.3.1. The clinical efficacy.

**Figure 2.**
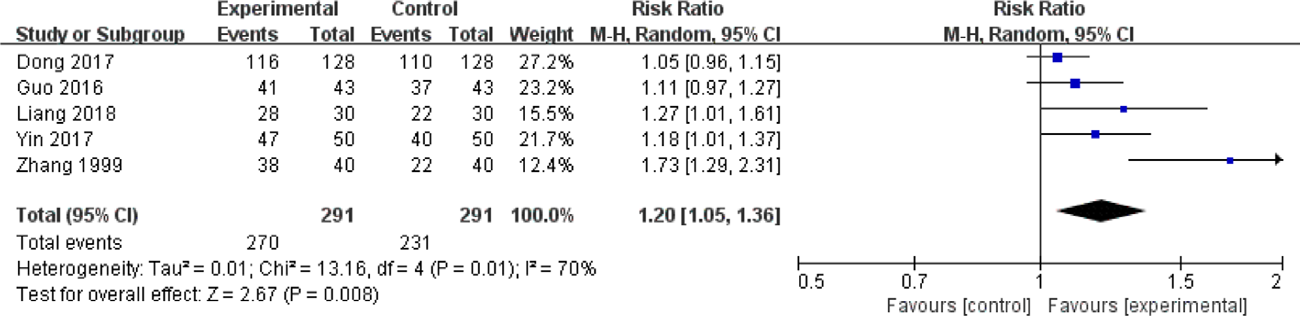
Forest plot of the clinical efficacy with a combination of vitamin A and conventional therapy versus conventional therapy alone for pneumonia in children.

Seven studies report the clinical efficacy, involving 582 children for pneumonia (treated group: 291 patients; control group: 291 patients). There is statistically significant heterogeneity (*P* = .01, *I*² = 70%); thus, a random-effect model is used. Meta-analysis shows that the total effective rate of experimental group is higher than that of control group. There is significant difference on the clinical efficacy between 2 groups (Z = 2.67, RR = 1.20, 95% CI: [1.05, 1.36], *P* = .008) (Fig. 2).

#### 3.3.2. The duration of fever.

**Figure 3.**
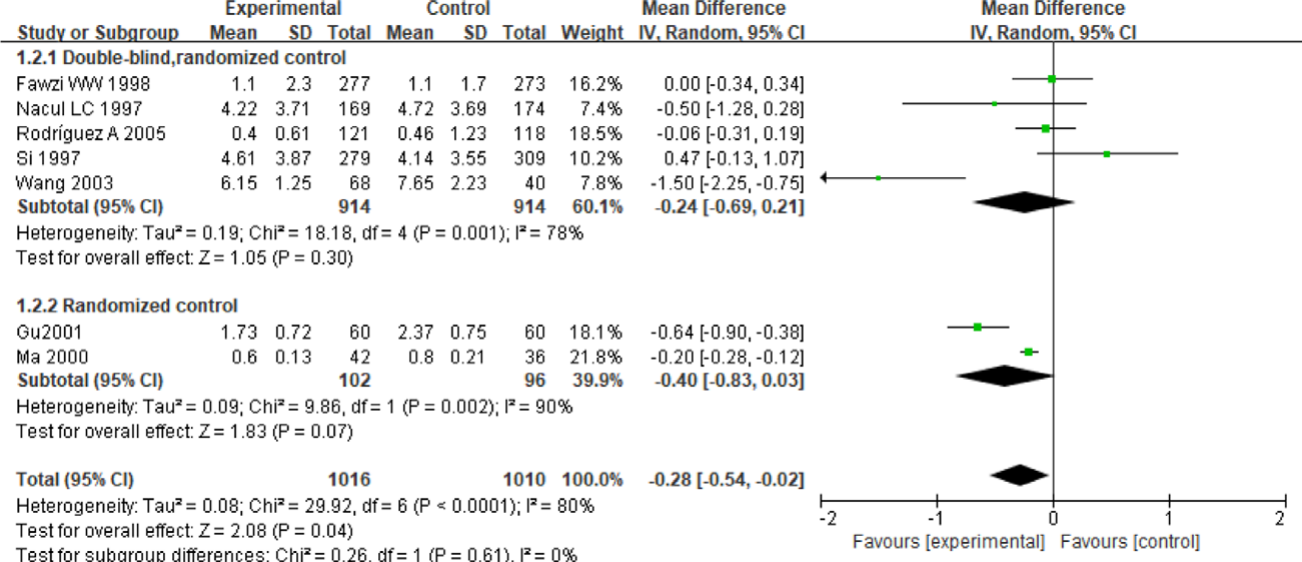
Forest plot of the duration of fever with a combination of vitamin A and conventional therapy versus conventional therapy alone for pneumonia in children.

Nine studies report the duration of fever, including 2026 children for pneumonia (treated group: 1016 patients; control group: 1010 patients). There is statistically significant heterogeneity (*P < *.0001, *I*² = 80%); thus, a random-effect model is used. The results of meta-analysis show that the duration of fever in experimental group is shorter than that in control group. There is significant difference on the duration of fever between 2 groups (Z = 2.08, MD = −0.28, 95% CI: [−0.54, −0.02], *P* = .04) (Fig. 3). Based on applying or not a double blind method in the RCTs, there is no statistical significance on subgroup analyzing between the experimental group and the control group.

#### 3.3.3. The time of lung rale disappearance.

**Figure 4.**
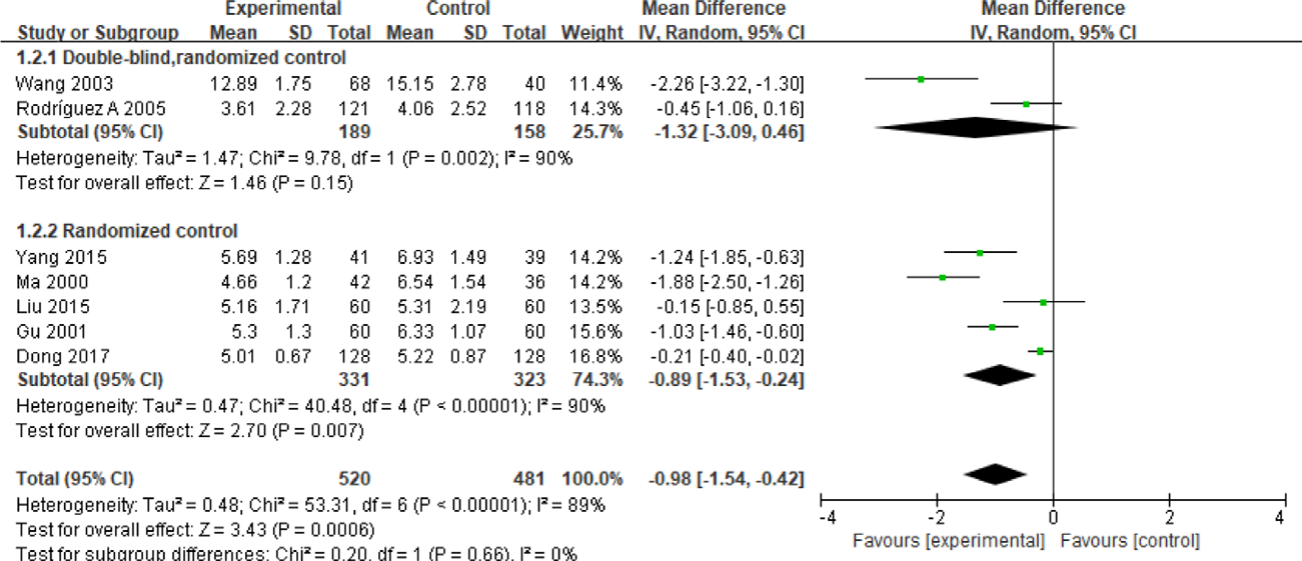
Forest plot of the time of lung rale disappearance with a combination of vitamin A and conventional therapy versus conventional therapy alone for pneumonia in children.

Ten studies report the time of lung rale disappearance, involving 1001 children for pneumonia (treated group: 520 patients; control group: 481 patients). There is statistically significant heterogeneity (*P < *.00001, *I*² = 89%); thus, a random-effect model is used. Meta-analysis shows that the disappearance time of pulmonary rales in the experimental group is shorter than that in the control group. There is significant difference on the time of lung rale disappearance between 2 groups (Z = 3.43, MD = −0.98, 95% CI: [−1.54, −0.42], *P = *.0006) (Fig. 4). Based on applying a double blind method in the RCTs, there is no statistical significance on subgroup analyzing between the experimental group and the control group. In the RCTs without a double blind method, however, there is a statistical significance (*P* = .007, *I*^2^ = 89%) between 2 groups.

#### 3.3.4. The time of choking milk disappearance.

**Figure 5.**
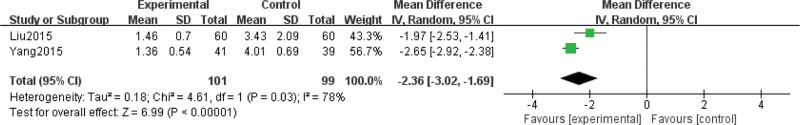
Forest plot of the time of choking milk disappearance with a combination of vitamin A and conventional therapy versus conventional therapy alone for pneumonia in children.

Three studies report the time of choking milk disappearance, involving 200 children for pneumonia (treated group: 101 patients; control group: 99 patients). There is statistically significant heterogeneity (*P* = .03, *I*² = 78%); thus, a random-effect model is used. The results of meta-analysis show that the disappearance time of choking milk in experimental group is shorter than that in control group. There is significant difference on the time of choking milk disappearance between 2 groups (Z = 6.99, MD = −2.36, 95% CI: [−3.02, −1.69], *P < *.00001) (Fig. 5).

#### 3.3.5. Negative time of chest X-ray.

**Figure 6.**
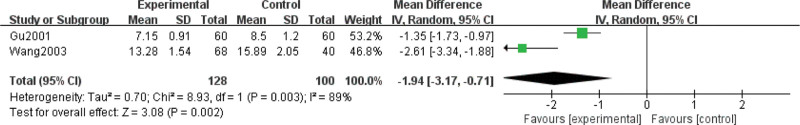
Forest plot of the negative time of chest X-ray with a combination of vitamin A and conventional therapy versus conventional therapy alone for pneumonia in children.

Two studies report the negative time of chest X-ray, including 228 children for pneumonia (treated group: 128 patients; control group: 100 patients). There is statistically significant heterogeneity (*P* = .003, *I*² = 89%); thus, a random-effect model is used. The results of meta-analysis show that the time of chest radiographs turning negative in experimental group is shorter than that in control group. There is significant difference on the negative time of chest X-ray between 2 groups (Z = 3.08, MD = −1.94, 95% CI: [−3.17, −0.71], *P* = .002) (Fig. 6).

#### 3.3.6. The duration of cough.

**Figure 7.**
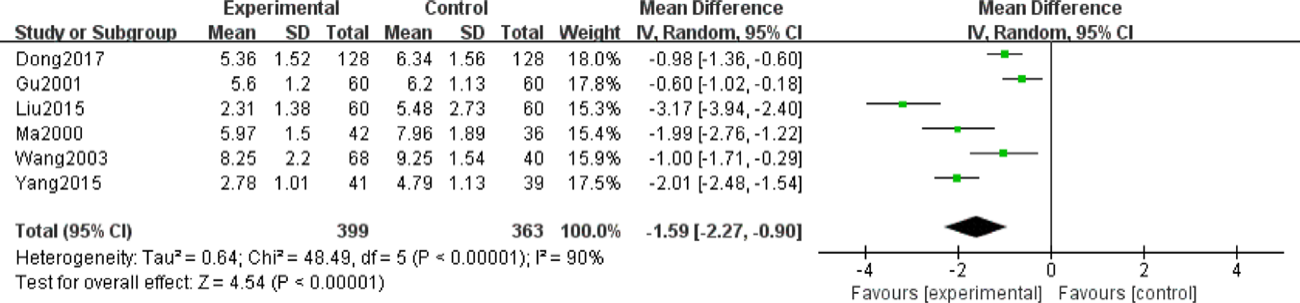
Forest plot of the duration of cough with a combination of vitamin A and conventional therapy versus conventional therapy alone for pneumonia in children.

Eight studies report the duration of cough, including 762 children for pneumonia (treated group: 399 patients; control group: 363 patients). There is statistically significant heterogeneity (*P < *.00001, *I*² = 90%); thus, a random-effect model is used. Meta-analysis results show that the duration of cough in the experimental group is shorter than that in the control group. There is significant difference on the duration of cough between 2 groups (Z = 4.54, MD = −1.59, 95% CI: [−2.27, −0.90], *P* < .00001) (Fig. 7).

#### 3.3.7. The hospitalization.

**Figure 8.**
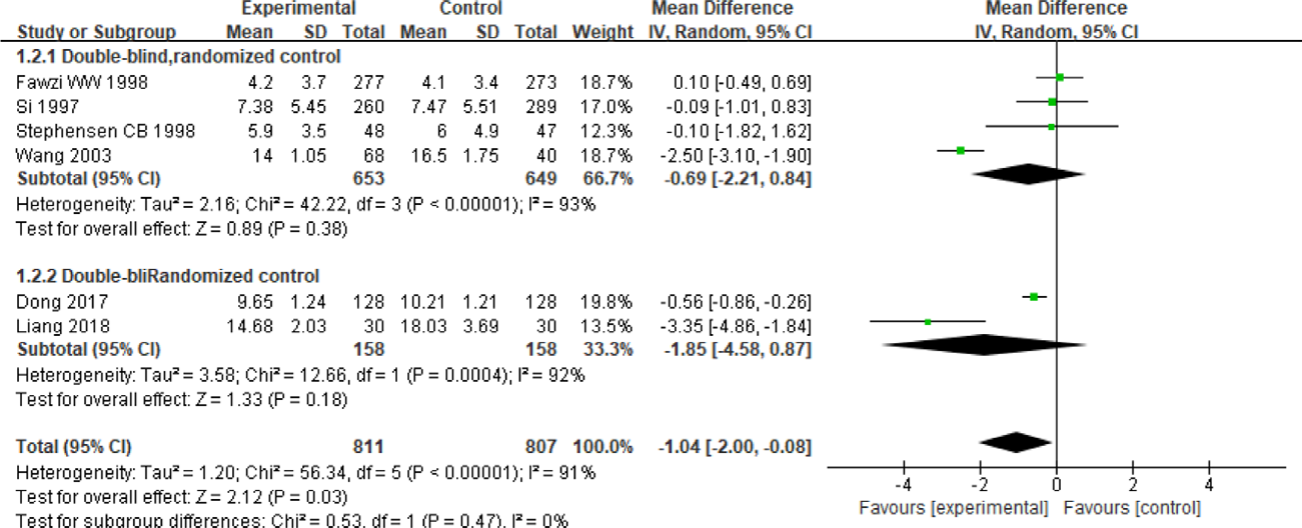
Forest plot of the time of hospitalization with a combination of vitamin A and conventional therapy versus conventional therapy alone for pneumonia in children.

Seven studies report the hospitalization, involving 1618 children for pneumonia (treated group: 811 patients; control group: 807 patients). There is statistically significant heterogeneity (*P < *.00001, *I*² = 91%); thus, a random-effect model is used. The results of meta-analysis show that the length of hospital stay in the experimental group is shorter than that in the control group. There is significant difference on the hospitalization between 2 groups (Z = 2.12, MD = −1.04, 95% CI: [−2.00, −0.08], *P* = .03) (Fig. 8). Based on applying or not a double blind method in the RCTs, there is no statistical significance on subgroup analyzing between the experimental group and the control group.

#### 3.3.8. The time of perilabial cyanosis disappearance.

**Figure 9.**
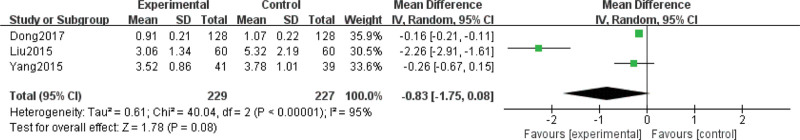
Forest plot of the time of perilabial cyanosis disappearance with a combination of vitamin A and conventional therapy versus conventional therapy alone for pneumonia in children.

Four studies report the time of perilabial cyanosis disappearance, involving 456 children for pneumonia (treated group: 229 patients; control group: 227 patients). There is statistically significant heterogeneity (*P < *.00001, *I*² = 95%); thus, a random-effect model is used. Meta-analysis show that the time of cyanosis disappearance in the experimental group is shorter than that in the control group. There is significant difference on the time of perilabial cyanosis disappearance between 2 groups (Z = 1.78, MD = −0.83, 95% CI: [−1.75, 0.08], *P* = .08) (Fig. 9).

#### 3.3.9. Mortality rate.

**Figure 10.**
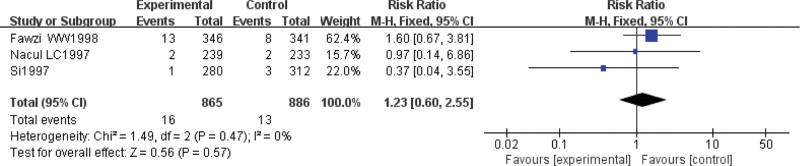
Forest plot of the mortality rate with a combination of vitamin A and conventional therapy versus conventional therapy alone for pneumonia in children.

Three studies (Si 1997, Nacul 1997, Fawzi 1998) report the mortality rate. There is no statistical heterogeneity (*P* = .47, *I*^2^ = 0%); thus, a fixed-effect model is used. There is no significant difference on the mortality rate between 2 groups (RR = 1.23, 95% CI: [0.60, 2.55], *P* = .57) (Fig. 10).

#### 3.3.10. Adverse events.

Fourteen studies mention no adverse event. Children with pneumonia were treated in strict accordance with the daily intake standard of vitamin A for children, and no adverse event is reported and discussed in the included studies. It indicate that vitamin A as an adjuvant therapy for pneumonia in children is safe and feasible.

## 4. Discussion

### 4.1. Analysis of vitamin A efficacy

The meta-analysis shows that vitamin A combined with conventional therapy has higher clinical efficacy than conventional therapy alone (RR = 1.11, 95% CI: [1.04, 1.19]). Vitamin A combined with conventional therapy is lower than conventional therapy alone on the duration of fever and cough (Z = 2.08, MD = −0.28, 95% CI: [−0.54, −0.02], *P* = .02; Z = 4.54, MD = −1.59, 95% CI: [−2.27, −0.90], *P <* .00001), negative time of chest X-ray (Z = 3.08, MD = −1.94, 95% CI: [−3.17, −0.71], *P* = .002), and the hospitalization (Z = 2.12, MD = −1.04, 95% CI: [−2.00, −0.08], *P* = .03), lung rale disappearance (Z = 3.43, MD = −0.98, 95% CI: [−1.54, −0.42], *P =* .0006), choking milk disappearance (Z = 6.99, MD = −2.36, 95% CI: [−3.02, −1.69], *P* < .00001), perilabial cyanosis disappearance (Z = 1.78, MD = −0.83, 95% CI: [−1.75, 0.08], *P* = .08). However, there is no significant difference on the mortality rate between 2 groups (RR = 1.23, 95% CI: [0.60, 2.55], *P* = .57). Therefore, vitamin A combined with conventional therapy for pneumonia in children can improve the clinical efficacy effectively than conventional therapy alone.

### 4.2. Limitations

There are a number of limitations to this study. Firstly, other indicators of heart function have not been searched comprehensively. Secondly, the sample size of included studies is small and different that may influence the results. Thirdly, multi-center and large scale RCTs are rare. Fourthly, we did not search the SCOPUS so maybe there is some literature bias. Finally, the description of randomization, allocation concealment and binding in the included studies is limited, which may cause selective bias and measurement bias. Thus, more high-quality studies are needed due to the limitations of sample size and methodological quality. And the mechanism of vitamin A as an adjuvant therapy for pneumonia in children still needs to be discussed.

### 4.3. Vitamin A and immune function

The World Health Organization estimates that 250 million children under the age of 5 suffer from VAD. Patients with VAD have higher mortality and morbidity from respiratory infections. Vitamin A is obtained through diet and is eventually converted to retinoic acid (RA) in the body, and RA is an active metabolite of vitamin A. RA is a key regulator of immune function and can alter the activity of both natural and adaptive immune cells.^[29]^ The role of vitamin A in the body is beneficial to adaptive immunity rather than inhibiting it. When vitamin A promotes functional CD4 + T cells, CD8 + T cells and B cells in the respiratory tract, can prevent cell death and immunopathology.^[30]^ VAD impairs natural immunity by impeding the normal regeneration of the mucosal barrier of infection damage and reducing the function of neutrophils, among others. Vitamin A is also essential for adaptive immunity. The above data explained from various aspects, vitamin A has enhanced and promoted the function of immune function. VAD increases the incidence of respiratory infections and may increase the risk or severity of bacterial infections. Vitamin A plays an important role in maintaining and increasing immune function.^[31]^

### 4.4. Application prospects

Vitamin A is required for maintenance of cell function for growth, epithelial integrity, red blood cell production, immunity and reproduction. VAD impairs body functions. It can increase risk of a range of problems, including susceptibility to infection, respiratory diseases, diarrhea, measles, stunting and anemia. All may lead to death. Vitamin A supplementation can reduce the incidence rate and mortality of children with diarrhea, and also reduce the incidence rate of measles in children.^[32]^ The incidence of respiratory tract infection in children with VAD is twice as high as that in normal children.^[33]^ β-carotene supplementation has a good effect on the recovery of pneumonia in children.^[34]^ A moderate dose of vitamin A supplementation has no effect on the duration of uncomplicated pneumonia in underweight or normal-weight children.^[14]^ Vitamin A had no significant effect on children with severe acute lower respiratory tract infection.^[35]^ Also, vitamin A is called an “anti-infective vitamin.”^[36]^ It plays a greater role in enhancing the recovery of infection than in preventing infection.^[37]^ Vitamin A is used for skin diseases, including cowhide disease, acne and light damage.^[38–41]^ The meta-analysis shows that vitamin A combined with conventional therapy is effective for pneumonia in children. And above studies also suggest that vitamin A combined with conventional therapy has significant effects in children with pneumonia. Thus, the results have certain clinical significance. Vitamin A and conventional therapy can greatly shorten the course of treatment. Moreover, vitamin A has wide sources, low price and few adverse events. Thus, vitamin as an adjuvant therapy for pneumonia in children is worth promoted.

## 5. Perspective

High morbidity and mortality of COVID-19 makes it as an urgent global health issue. Vitamin A is believed to form the first line of defense against pathogens by playing a role in stratification, keratinization, differentiation and functional maturation. It participates in the formation of healthy mucinous layer and enhances antigen-specific immune function. RA, the active form of vitamin A, regulates the innate immune system.^[42]^ Research has shown that VAD can reduce the body’s resistance to COVID-19 virus infection.^[43]^ It plays an important role in the recovery of lung tissue after injury. Bioinformatics identifies possible targets, therapeutic pathways and pharmacological functions for vitamin A against COVID-19 infection.^[44]^ Vitamin A might treat the COVID-19 in terms of immunomodulatory, antiviral, associated cellular protection, and anti-inflammatory effects.

## 6. Conclusion

Vitamin A contributes to relieve the clinical symptoms and signs, and shorten the hospitalization.

## Author contributions

All authors contributed to the design and concept, performed the literature searches, wrote the manuscript and critiqued the successive versions, and approved the final manuscript. HEB coordinated the effort and integrated the sections and comments.

**Conceptualization:** Wenli Zhao, Hongwu Wang, Ye Zhao, Huaien Bu.

**Data curation:** Ruoxi Li, Wenli Zhao, Ye Zhao, Huaien Bu.

**Formal analysis:** Ruoxi Li, Huaien Bu.

**Funding acquisition:** Hongwu Wang.

**Investigation:** Wenli Zhao, Huaien Bu.

**Methodology:** Wenli Zhao, Hongwu Wang, Maeda Toshiyoshi, Huaien Bu.

**Project administration:** Huaien Bu.

**Resources:** Maeda Toshiyoshi, Huaien Bu.

**Software:** Maeda Toshiyoshi, Huaien Bu.

**Supervision:** Hongwu Wang, Huaien Bu.

**Writing – original draft:** Ruoxi Li, Wenli Zhao, Maeda Toshiyoshi, Ye Zhao, Huaien Bu.

**Writing – review & editing:** Maeda Toshiyoshi, Ye Zhao, Huaien Bu.

## Acknowledgments

The authors thank Dr Bin Wang for assistance with data extraction.
